# Screening differential circular RNA expression profiles reveals hsa_circ_0004018 is associated with hepatocellular carcinoma

**DOI:** 10.18632/oncotarget.16881

**Published:** 2017-04-06

**Authors:** Liyun Fu, Ting Yao, Qingqing Chen, Xiaoyan Mo, Yaoren Hu, Junming Guo

**Affiliations:** ^1^ Department of Hepatology, Ningbo No. 2 Hospital and The Affiliated Hospital, Medical School of Ningbo University, Ningbo 315010, China; ^2^ Department of Biochemistry and Molecular Biology, Zhejiang Key Laboratory of Pathophysiology, Medical School of Ningbo University, Ningbo 315211, China

**Keywords:** hsa_circ_0004018, circular RNA, hepatocellular carcinom, microRNA, biomarker

## Abstract

Circular RNAs (circRNAs) have been emerged as an indispensable part of endogenous RNA network. However, the expression significance of circRNAs in hepatocellular carcinoma (HCC) is rarely revealed. The aim of this study was to determine the circRNA expression profile in HCC, and to investigate their clinical significances and relevant mechanisms for cancer progression. The global circRNA expression profile in HCC was measured by circRNA microarray. Levels of one representative circRNAs, hsa_circ_0004018, were confirmed by real-time reverse transcription-polymerase chain reaction. The expression levels of hsa_circ_0004018 in HCC were significantly lower compared with para-tumorous tissue (*P*<0.001). Our data further showed that lower expression of hsa_circ_0004018 was correlated with serum alpha-fetoprotein (AFP) level, tumor diameters, differentiation, Barcelona Clinic Liver Cancer stage and Tumor-node-metastasis stage. More importantly, we detected liver tissues from chronic hepatitis, cirrhosis and HCC patients; and found that hsa_circ_0004018 harbored HCC-stage-specific expression features in diverse chronic liver diseases (*P*<0.001). The area under receiver operating characteristic curve was up to 0.848 (95% CI=0.803–0.894, *P*<0.001). The sensitivity and specificity were 0.716 and 0.815, respectively. Finally, hsa_circ_0004018 might be involved in cancer-related pathways via interactions with miRNAs.

## INTRODUCTION

Globally, hepatocellular carcinoma (HCC) is the most common type of hepatic malignancies, accounting for approximately 90% of primary liver cancer. It ranks as the second most significant cause of cancer-related deaths in men, 50% of the cases and deaths occurred in China [1]. It is disappointing that most HCC patients were diagnosed at advanced stages with metastasis, missing the best opportunity for curative therapy, such as resection, transplantation or ablation [2]. The early diagnosis is urgent for prognosis of HCC [3]. The risk of HCC increases with liver fibrosis stages [4]. Many studies revealed that the intimate relationship between cirrhosis and HCC [5–8]. In general, anyone with cirrhosis should be screened for HCC [9].

In recent years, circular RNAs (circRNAs) have emerged as a new star in noncoding RNA (ncRNA) world, representing a class of endogenous RNAs existing in mammalian cells and featuring stable structure and high cell-type-specific, tissue-specific and developmental-specific expression [10]. By interacting with microRNAs (miRNAs) or other molecules, circRNAs regulate gene expression at the transcriptional or post-transcriptional level [10, 11]. Compared to linear RNAs, circRNAs have the outstanding feature of non-canonical splicing without a free 3’ and 5’end, which enables them to resist RNA exonucleases [10–12]. As a result, they might be suitable as potential biomarkers and targets for novel therapeutic approaches for human diseases. Recently, researchers have found that circRNAs are linked to several cancers, such as gastric cancer, colorectal cancer, HCC, pancreatic ductal adenocarcinoma and ovarian cancer [13–16].

Since the global circRNA expression profile in HCC is not fully uncovered, in the present study, we explored the circRNA expression profile in HCC. We identified 527 differentially expressed circRNAs (including 174 upregulated and 353 downregulated genes) in HCC tissues compared with para-tumorous tissues. And then, we focused on hsa_circ_0004018, one of the most downregulated circRNAs in microarray detection (fold change=294.86, *P* value=4.7164E-07). It is transcribed from *SMYD4* (SET and MYND domain containing 4) on chromosome 17.

## RESULTS

### Overview of circRNA profiles in HCC tissues

**Figure 1:**
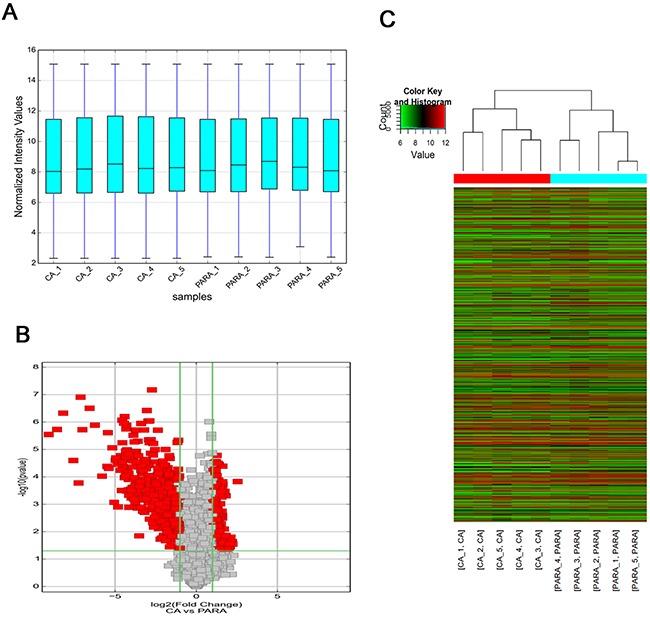
Overview of the microarray signatures **(A)** The boxplot view is used to compare the distributions of expression values for the samples in an experiment after standardization. After standardization, the distributions of log2 ratios among ten samples are nearly the same (CA: HCC; PARA: para-tumorous tissue). **(B)** Volcano plot of the differentially expressed circRNAs. The vertical lines correspond to 2.0-fold up and down, respectively, and the horizontal line represents a p-value of 0.05. The red point in the plot represents the differentially expressed circRNAs with statistical significance. **(C)** The result of hierarchical clustering shows a distinguishable circRNA expression profiling among samples. Each column represents the expression profile of a tissue sample, and each row corresponds to a circRNA. ‘‘Red’’ indicates higher expression level, and ‘‘green’’ indicates lower expression level.

Our microarray analysis revealed the circRNA expression profiles in five paired human HCC and para-tumorous tissues (GEO No. 94508: https://www.ncbi.nlm.nih.gov/geo/query/acc.cgi?acc=GSE94508). The box plot is a direct way to rapidly visualize the distributions of a dataset for the circRNAs profiles. After normalization, the distributions of log2 ratios among ten samples are nearly the same (Figure 1A). Differentially expressed circRNAs with statistical significance (fold changes ≥2.0 and *P* <0.05) between groups were identified by volcano plot filtering (Figure 1B). The circRNA expression patterns between HCC tissues and para-tumorous tissues were found to be significantly different (Figure 1C). A total of 527 circRNAs, whose expression change were more than twofold, were found. Among them, 174 and 353 were up and down expressed in tumor tissues, respectively. The number of down-expressed circRNAs was bigger than that of up-expressed circRNAs. We selected hsa_circ_0004018 for further study.

### Hsa_circ_0004018 was downregulated in HCC tissues and HCC cell lines

**Figure 2:**
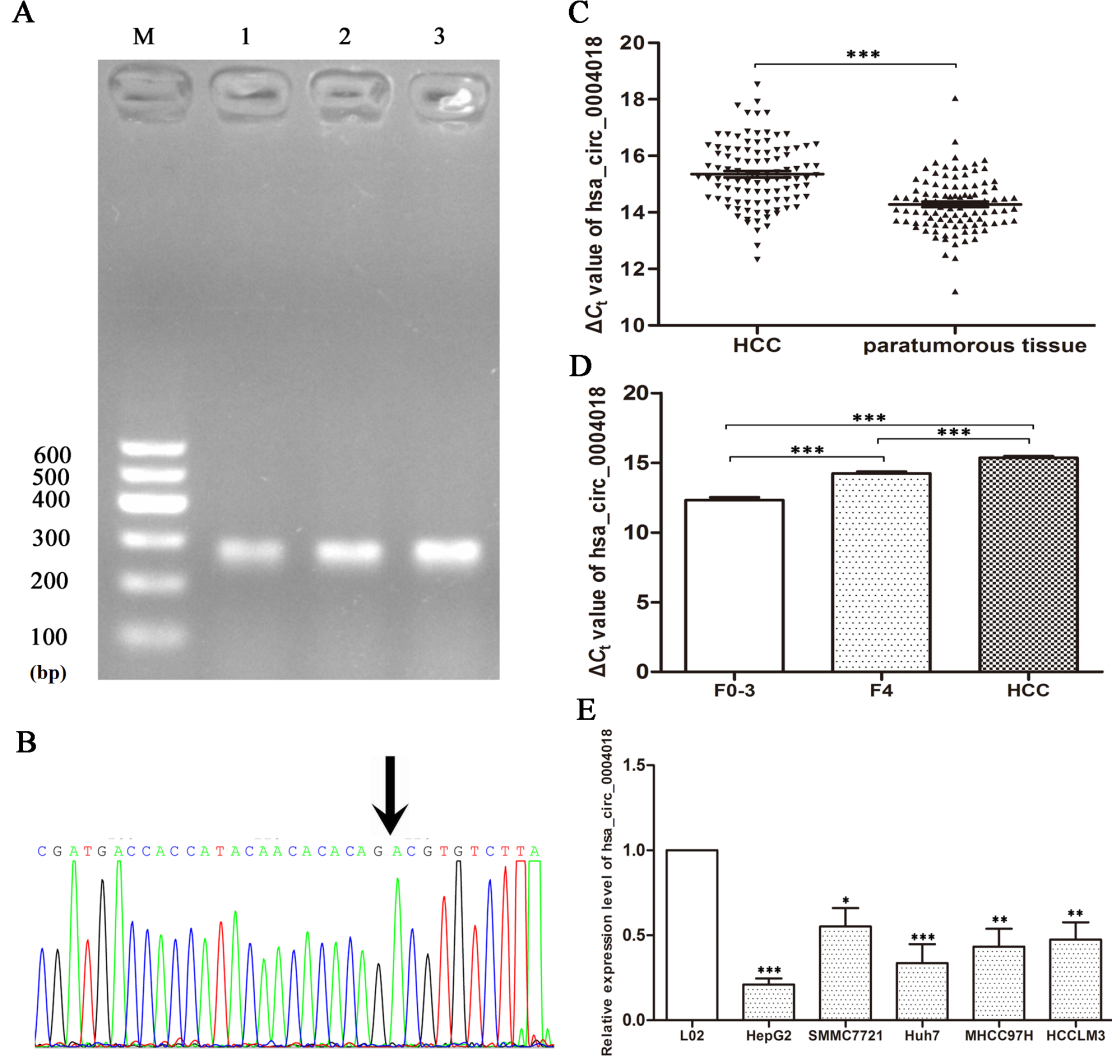
Hsa_circ_0004018 expression features **(A)** Electrophoresis of qRT-PCR products. Lane M is DL600 Marker, lanes 1-3 are qRT-PCR products. **(B)** Validation of hsa_circ_0004018 splicing junction site through sequencing of its qRT-PCR product. (**C)** Decreased expression of hsa_circ_0004018 in HCC tissues. Real-time RT-PCR was used to determine the expression level. The Δ*C*t value was determined by subtracting the *C*t value of GAPDH from the *C*t value of circRNA. Larger Δ*C*t value indicates lower expression. (*n*=102, *P*<0.001). **(D)** Hsa_circ_0004018 expression level among liver tissues from HCC (*n*=102), cirrhosis (*n*=63), and chronic hepatitis (*n*=66). **(E)** Expression of hsa_circ_4018 in HCC cell lines and normal hepatic cell line L02. Data are means± SD.

We used qRT-PCR method to measure the hsa_circ_0004018 expression levels in liver tissues from chronic hepatitis, cirrhosis, HCC and para-tumorous tissues. The head-to-tail splicing junction of hsa_circ_0004018 was confirmed by sequencing of the product of qRT-PCR (Figure 2B), which was consistent with that from the circbase (http://www.circbase.org/cgi-bin/singlerecord.cgi?id=hsa_circ_0004018). As is shown in Figure 2C, hsa_circ_0004018 was down-regulated in 92.2% (94/102) of HCC tissues (*P*<0.001). As shown in Figure 2E, compared with human normal hepatic cell line L02, we found that the levels of hsa_circ_0004018 were down-regulated in HCC cell lines, HepG2 (*P*<0.001), SMMC7721 (*P*<0.05), Huh7 (*P*<0.01), MHCC97H (*P*<0.01), and HCCLM3 (*P*<0.01).

### Correlations between hsa_circ_0004018 expression levels and clinicopathological parameters in HCC patients

Then, we analyzed the relationship between the expression levels of hsa_circ_0004018 and clinicopathological factors of patients with HCC. As Table 1 indicates, hsa_circ_0004018 level was correlated with serum alpha-fetoprotein (AFP) level, tumor diameters, differentiation, Barcelona Clinic Liver Cancer (BCLC) stage and Tumor-node-metastasis (TNM) stage.

**Table 1:** The relationship between hsa_circ_0004018 expression levels (Δ*C*t) in cancer tissues and clinicopathological factors of patients with HCC

Characteristics	No. of patients	Percent of patients (%)	Mean±SD	*P* value
Age (years)				0.236
≥50	78	76.5	15.27±1.13	
<50	24	23.5	15.60±1.18	
Gender				0.916
Male	90	88.2	15.35±1.19	
Female	12	11.8	15.38±0.85	
Family history				0.144
Positive	65	63.7	15.47±1.19	
Negative	37	36.3	15.14±1.06	
Diabetes mellitus				0.685
Yes	14	13.7	15.21±1.44	
No	88	86.3	15.38±1.10	
Encapsulation				0.421
Yes	66	64.7	15.28±1.14	
No	36	35.3	15.48±1.16	
Tumor number				0.613
Single	72	70.6	15.39±1.13	
Multiple	30	29.4	15.26±1.21	
Diameter (cm)				0.045
≥5	40	39.2	15.64±1.13	
<5	62	60.8	15.17±1.13	
Differentiation				0.006
Poor	29	28.4	15.90±1.26	
Moderate and well	73	71.6	15.14±1.03	
Microvascular invasion				0.119
Positive	41	41.4	15.57±1.38	
Negative	58	58.6	15.20±0.92	
BCLC stage				0.040
A	45	44.1	15.09±1.04	
B+C+D	57	55.9	15.56±1.20	
TNM stage				0.029
I	43	42.2	15.04±0.96	
II	29	28.4	15.39±1.27	
III+IV	30	29.4	15.76±1.18	
HbsAg				0.938
Negative	15	14.9	15.38±1.17	
Positive	86	85.1	15.35±1.16	
Serum AFP				0.027
>20	61	62.2	15.52±1.15	
≤20	37	37.8	15.00±1.09	
Serum AKP				
>95	53	52.5	15.23±1.12	0.226
≤95	48	47.5	15.51±1.16	
Serum GGT				0.282
>50	53	52.0	15.47±1.27	
≤50	49	48.0	15.22±1.00	

### Dynamic changes of hsa_circ_0004018 levels in groups from chronic hepatitis to cirrhosis to HCC

To confirm the results of hsa_circ_0004018 in HCC patients (Figure 2B), we selected cirrhosis and the chronic hepatitis biopsy tissues. Intriguingly, we revealed that the levels of hsa_circ_0004018 in HCC tissues were significantly lower than those of cirrhosis (F=4) (*P*<0.001); and its levels in cirrhosis tissues were significantly lower than those in chronic hepatitis tissues (F=0-3) (*P*<0.001). Hsa_circ_0004018 expression levels exhibited HCC-stage-specific characteristics (Figure 2D).

### Potential diagnostic values of hsa_circ_0004018

**Figure 3:**
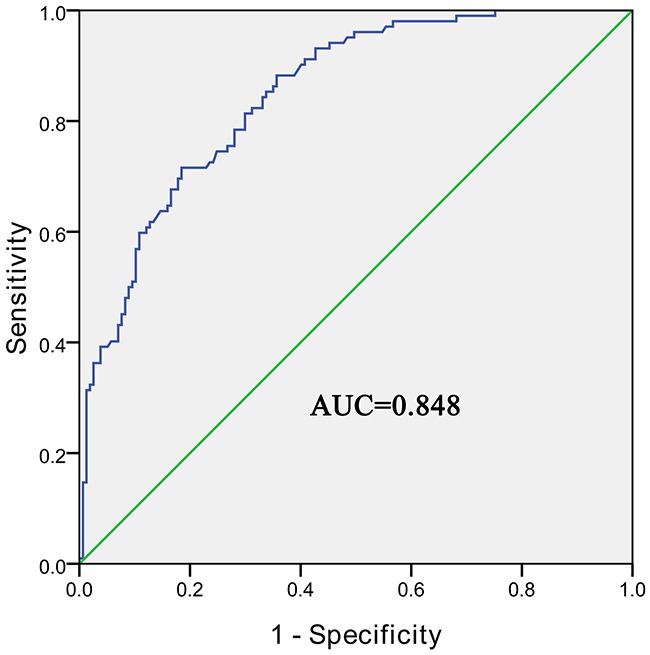
The ROC curve of using hsa_circ_0004018 as a biomarker *P*<0.05.

We then used the ROC curve to investigate the diagnostic value of hsa_circ_0004018 in distinguishing HCC tissues from para-tumorous and chronic hepatitis liver tissues. We found that the area under the ROC curve (AUC) was 0.848 (95% CI=0.803–0.894, *P*<0.001, Figure 3). The sensitivity and specificity were 0.716 and 0.815, respectively. The Youden index was 0.531.

### Annotation for circRNA/miRNA interaction

**Figure 4:**
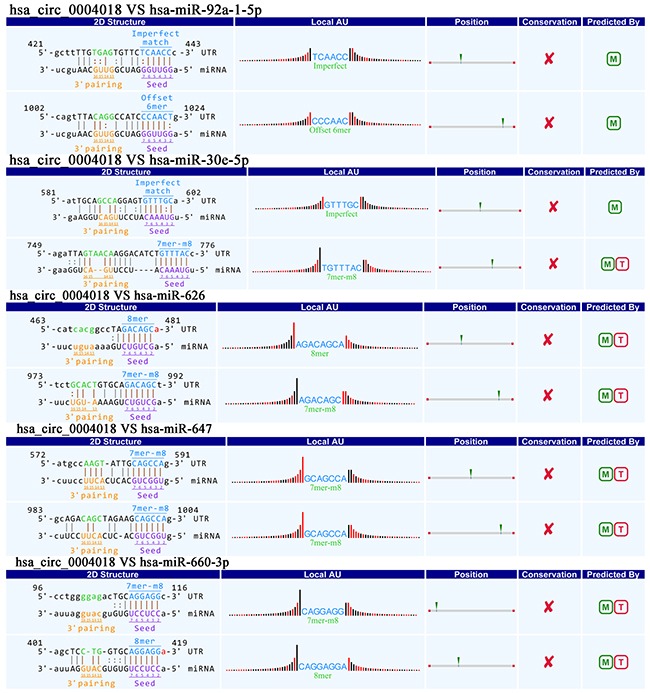
Prediction for hsa_circ_0004018/miRNA interactions

To excavate functions of circRNAs, we investigated potential miRNAs binding with circRNAs. Hsa_circ_0004018 was annotated in detail via Arraystar's home-made miRNA target prediction software based on TargetScan and miRanda, and was showed to harbor hsa-miR-30e-5p, hsa-miR-647, hsa-miR-92a-1-5p, hsa-miR-660-3p and hsa-miR-626 by miRNAs seed sequence matching (Figure 4).

### Prediction of circRNA-miRNA-mRNA associations

**Figure 5:**
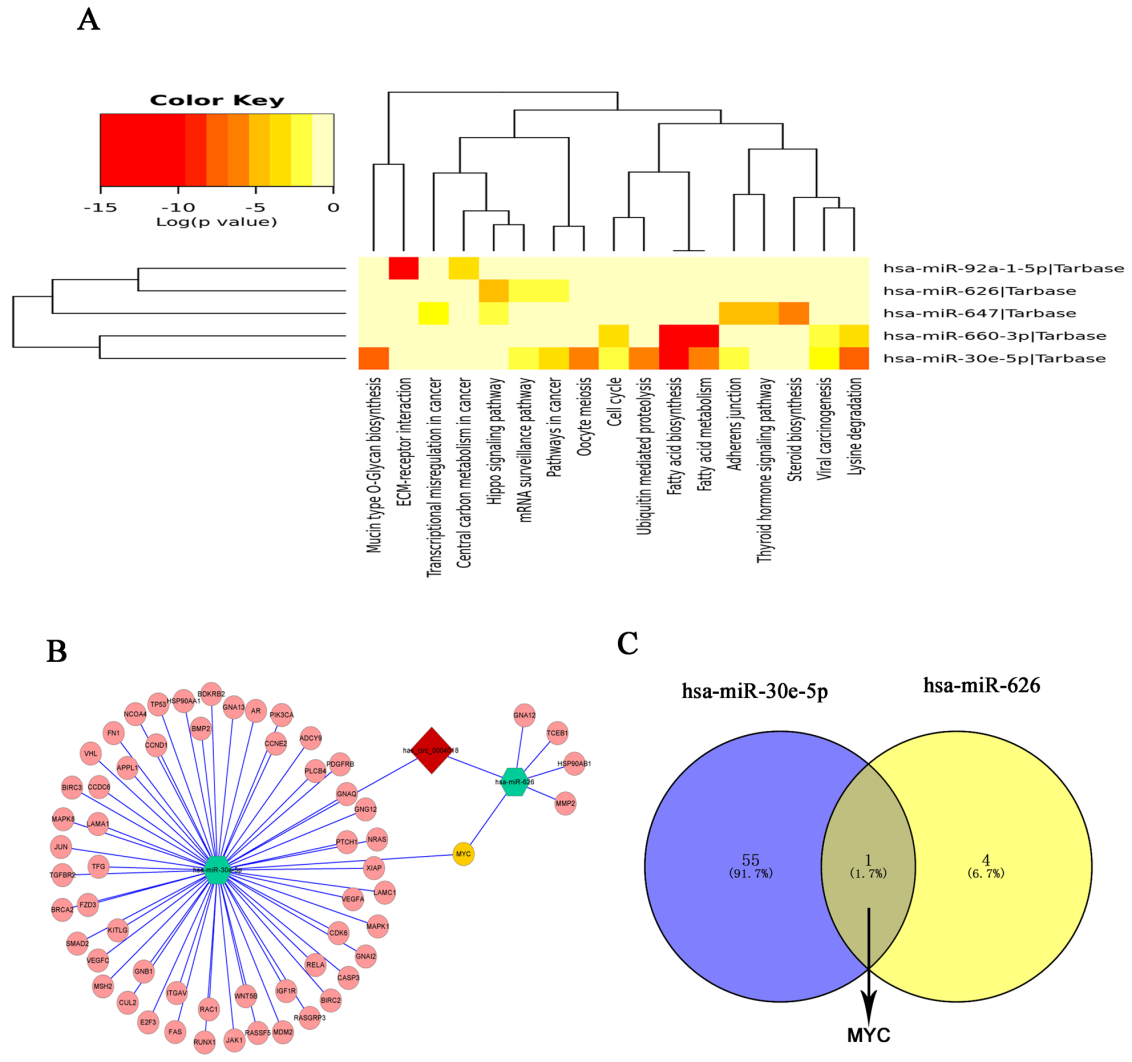
Prediction for hsa_circ_0004018/miRNA-mediated pathways **(A)** The heatmap reveals statistically significant correlations between hsa-miR-30e-5p, hsa-miR-647, hsa-miR-92a-1-5p, hsa-miR-660-3p and hsa-miR-626 and their mediated pathways by *P*-value (log scaled). Red represents high significance. **(B)** A network comprising has_circ_0004018 (red diamond), hsa-miR-30e-5p, hsa-miR-626(green hexagon), their target miRNAs (pink nodes) and MYC (yellow node) is presented. **(C)** Venn diagram revealed the gene showed MYC are the common target gene of hsa-miR-30e-5p and hsa-miR-626.

In order to explore the molecular mechanism of hsa_circ_0004018, the hsa_circ_0004018-miRNA-mRNA axis in cancer-related pathways was predicted. DIANA-miRPath determined all the candidate miRNAs that are involved in possible pathways by *p*-value cutoff at 0.05. As Figure 4 shown, hsa_circ_0004018 could harbor five miRNAs seed sequence matching. Then, DIANA-miRPath analysis revealed that both hsa-miR-30e-5p and hsa-miR-626 were associated with cancer-related pathways (Figure 5A). After that, through TarBase7.0, *MYC* are hsa-miR-30e-5p and hsa-miR-626 validated common target genes. A network of hsa_circ_0004018-hsa-miR-30e-5p/hsa-miR-626-mRNA interaction was delineated using Cytoscape (Figure 5B); and the Venn diagram revealed the gene intersection (Figure 5C). These findings suggest that hsa_circ_0004018-miR-30e-5p/miR-626-MYC axes might play a part in HCC carcinogenesis and metastasis.

## DISCUSSION

CircRNAs have been recently found to be pervasively transcribed in the genome, and to be associated with human diseases. Studies have revealed that circRNAs express in a complex tissue-specific, cell-type-specific or developmental-stage specific manner [10, 17]. In present study, we investigated circRNAs expression profile of HCC using the microarray analysis. We found that the circRNAs expression levels in HCC samples were different from paracancerous tissues. The microarray expression profiles exhibited that 174 up-regulated circRNAs and 353 down-regulated circRNAs were significantly differentially expressed in five paired HCC samples; and showed hsa_circ_0004018's expressive feature in HCC for the first time. Compared with matched non-tumorous tissues, the expression level of hsa_circ_0004018 in HCC patients significantly decreased (Figure 2C). In addition, the levels of has_circ_0003570 in HCC cell lines were lower than those in L02. To get a closer insight into the clinical process of HCC, we compared the differences among liver tissues from patients with chronic hepatitis, cirrhosis, and HCC. Chronic hepatitis is well known as a dynamic disease which experience variable periods of immune activity versus quiescence that lead to the development of cirrhosis and liver cancer in a part of patients [18]. Liver fibrosis is a reversible wound-healing response to any etiology of chronic hepatic injuries. The risk of liver-related mortality increases exponentially with the increase in fibrosis stage [19, 20]. Our results showed hsa_circ_0004018 possessed a HCC-stage-specific expression feature (Figure 2D). Furthermore, lower expression of hsa_circ_0004018 was associated with serum AFP level, tumor diameters, differentiation, BCLC stage and TNM stage (Table 1). Hsa_circ_0004018 is transcribed from *SMYD4*, which is a potential tumor suppressor and plays critical roles in carcinogenesis, the development of heart and skeletal muscle, and hematopoiesis [21–23].

Recently, several laboratories have revealed thousands of circRNAs [10, 17, 24, 25]. In comparison to their cognate linear isoforms, some circRNAs’ expression levels are even above 10-fold. Most importantly, because of the fact that circRNAs are free of ends, they are resistant to digestion with conventional RNA degradation pathways [25, 26]. They are appealed as biomarkers for disease diagnosis and monitoring. For example, Li et al. found that hsa_circ_002059 could be used as a biomarker in the diagnosis of gastric cancer, for the first time [27]. Zhong et al. found that circTCF25 might be a new promising marker for bladder cancer [28]. Qin et al revealed that hsa_circ_0001649 is potential novel biomarker for HCC [29]. AFP, most used serum test for HCC surveillance and diagnosis, has been challenged in recent years [30]. A lot of clinical research showed diagnostic sensitivity is around 60% even with 10–20μg/L as AFP cutoff value [31]. Our results showed hsa_circ_0004018 is a valuable biomarker for HCC monitoring, the sensitivity and specificity of which were 0.716 and 0.815, respectively. And most importantly, the sensitivity of hsa_circ_0004018 is superior to that of AFP.

The function and mechanism of most circRNAs are not completely known [32, 33]. Recent studies have reported that circRNAs could function as a miRNA sponge to regulate the gene expression in cancers [11, 25, 34–36]. Competing endogenous RNA (ceRNA) described a complex post-transcriptional regulatory network mediated by miRNAs: by sharing one or more miRNA response elements (MREs), protein-coding and noncoding RNAs compete for binding to miRNAs and then adjust each other's expression [37]. CeRNAs are widely implicated in many biological processes [38]. A circular isoform of antisense noncoding RNA in the INK4 locus (ANRIL) has been revealed to correlate with cyclin-dependent kinase inhibitor 2/ alternate open reading frame (INK4/ARF) expression and impact the development of atherosclerosis [39]. *CDR1* antisense RNA (Cdr1as, also known as CiRS-7), one of representative circRNAs, was recently shown to harbor 76 miR-7 binding sites and to influence many diseases including HCC, diabetes, prion disorders and cancers [40–42]. Xu et al. firstly revealed the effects of the Cdr1as/miR-7 axis on insulin secretion through targeting *Myrip* and *Pax6*, which may become a new target for improving β cell function in diabetes [43]. In benzo[a]pyrene-induced carcinogenicity, via high-throughput data integration of RNA–miRNA–circRNA, Caiment et al. revealed novel insights into circRNAs’ mechanism study [13]. With bioinformatical methods, we found that hsa_circ_0004018 have MREs of hsa-miR-30e-5p, hsa-miR-647, hsa-miR-92a-1-5p, hsa-miR-660-3p and hsa-miR-626. And combined with DIANA-miRPath, we furtherly revealed that hsa_circ_0004018 might play important roles in HCC via hsa_circ_0004018-miR-30e-5p/miR-626-MYC axes. The oncogenic transcription factor c-Myc is one of famous oncogene, and has been revealed pathologically to be activated in many human cancers [44, 45]. And As Figure 5A showed hsa-miR-647 exhibited relationship with transcriptional misregulation in cancer; hsa-miR-92a-1-5p exhibited relationship with central carbon metabolism in cancer; hsa-miR-626 and hsa-miR-647 exhibited relationship with Hippo signaling pathway; hsa-miR-660-3p and hsa-miR-30e-5p exhibited relationship with viral carcinogenesis. These all showed hsa_circ_0004018 is closely related with malignancies.

In conclusion, as one of circRNAs, hsa_circ_0004018 was lowly expressed in HCC. At the same time, hsa_circ_0004018 showed HCC-stage-expressive characteristics from chronic hepatitis to cirrhosis and to HCC. These indicate that hsa_circ_0004018 not only might be a potential biomarker for the diagnosis of HCC, but also play a role in the carcinogenesis and metastasis of HCC. And further detailed mechanism studies underlying hsa_circ_0004018 are being carried out in our laboratory.

## METHODS

### Patients and specimens

The total of 102 HCC patients, who underwent surgeries at three medical centers (Ningbo No. 2 Hospital, Ningbo Lihuili Hospital and Ningbo Yinzhou Peoples’ Hospital, China) from March 2013 to December 2016, were included in this study. The para-tumorous tissues were obtained from 1 cm away from the edge of the HCC; and there were no obvious tumor cells. The diagnosis of HCC was confirmed by histological examination. Staging was determined by the BCLC staging system [30] and American Joint Committee on Cancer criteria [46]. Patients with HCC who had prior treatment of their tumor or history of other solid tumors were excluded in this study.

Other liver tissues were collected from 55 cases of chronic hepatitis patients from September 2013 to December 2016 in Ningbo No. 2 Hospital through liver biopsy under guided ultrasound. Fibrosis stage was assessed by the METAVIR scoring system (grades the stage of histological activity on a four-point scale: no activity=A0, mild activity=A1, moderate activity=A2, and severe activity=A3; grades the stage of fibrosis on a five-point scale: no fibrosis=F0, portal fibrosis without septa= F1, portal fibrosis with rare septa=F2, numerous septa without cirrhosis=F3, cirrhosis=F4) [47]. In all adjacent non-tumorous tissues, 74 cases of para-tumorous liver tissues were pathological diagnosis; among them, 60 cases showed LC (F=4) and 14 cases showed CH (F=0-3). Among 55 cases of chronic liver tissues by ultrasound guided, 3 cases showed LC (F=4) and 52 cases showed CH (F=0-3). Cirrhosis tissues group consisted of 60 para-tumorous liver tissues and 3 liver tissues of chronic hepatitis patients if METAVIR scoring was F4. Chronic hepatitis group consisted of 14 para-tumorous liver tissues and 52 liver tissues of chronic hepatitis patients METAVIR scoring was F0-3 with any A grade.

After being obtained, tissue samples were immediately soaked in RNA fixer Reagent (Bioteke, Beijing, China) and stored at −80 °C until used. Histology was independently assessed by two experienced pathologists who were blinded to the clinical data. This study was approved by the Human Research Ethics Committee from Ningbo University. Informed consent was obtained from all patients.

### Cell culture

HCC cell lines, HepG2, Huh7, SMMC-7721, MHCC97H and HCCLM3, and human normal hepatic cell line L02 were cultured with RPMI 1640 Medium (Life Technologies, Carlsbad, CA, USA) containing 10% fetal bovine serum in a humidified atmosphere of 5% CO_2_.

### RNA extraction

Total RNA was extracted by TRIzol reagent (Invitrogen, Karlsruhe, Germany) in accordance with the manufacturer's instructions. Concentration and purity of total RNA samples were measured by the Smart Spec Plus spectrophotometer (Bio-Rad, Hercules, CA). If the ratio of A260/A280 was 1.8–2.0, RNA was used for further experiments.

### Microarray data analysis

The microarray detection was performed by KangChen Bio-tech (Shanghai, China) under the guidance of the experiment workflow [15]. The circRNA/microRNA (miRNA) interaction was predicted using Arraystar's home-made miRNA target prediction software based on TargetScan and miRanda [15].

### Reverse transcription

The cDNA was generated using the GoScript Reverse Transcription (RT) System (Promega, Madison, WI) following the manufacturer's instructions. Briefly, 2μg total RNA, 1μl random primer, 1μl oligo(dT)15 primer, 2μl MgCl_2_, 4μl GoScript 5×reaction buffer, 1μl nucleotide mix, 0.5μl recombinant RNasin ribonuclease, and 1μl GoScript reverse transcriptase were added in the system and then incubated at 42 °C for 1h. RT reaction and no-template control were run at the same time.

### Quantitative real-time PCR

Quantitative polymerase chain reaction (qPCR) was performed using the GoTaq qPCR Master Mix (Promega) on an Mx3005P real-time PCR System (Stratagene, La Jolla, CA) in the light of the protocol. Outward facing Primers were designed with Primer3 (http://www-genome.wi.mit.edu) and synthesized by Sangon Biotech (Shanghai, China). Their sequences were as follows: for hsa_circ_0004018 (target gene) 5’- TCAACCTTTTGCCCCACACT-3’ and 5’- AAGACACGTCTGTGTGTTGT-3’; and for glyceraldehyde 3-phosphate dehydrogenase (GAPDH, reference gene), 5’-TCGACAGTCAGCCGCATCTTC TTT-3’ and 5’-ACCAAATCCGTTGACTCCGACCTT-3’. Real-time PCR was done in triplicate. The amplification specific was confirmed by melting curve analysis. The data from qRT-PCR were analyzed by the Δ*C*t method and the 2^−ΔΔ*C*t^ method. All results are expressed as the means±SD. All of assays were performed in a blinded fashion.

### Electrophoresis and sequencing of qRT-PCR products

To prove the qRT-PCR products, 1.5% agarose gel electrophoresis was used. Afterwards, based on the manufacturer's instructions, the qRT-PCR product of hsa_circ_0004018 was first purified by using a UNIQ-10 PCR Product Purification Kit, and cloned into the pUCm-T vector (Sangon Biotech). After that, DNA sequencing was performed by Sangon Biotech Co., Ltd.

### Liver function and serological tumor marker analysis

Liver function including total protein (TP), albumin, aspartate transaminase (AST), alanine aminotransferase (ALT), alkaline phosphatase (AKP), gamma glutamyl transferase (GGT), and total bilirubin was measured by Olympus AU 2700 automatic biochemical analyzer with original kits (Olympus, Tokyo, Japan). AFP was measured with an Elecsys 2010 machine (Roche Diagnostics, Basel, Switzerland).

### Prediction for circRNA-miRNA-mRNA pathways

The miRNA pathway was carried out based on DIANA-miRPath [48]. All of miRNA gene targets are experimentally validated (derived from TarBase 7.0) [49]. The graph of the circRNA/miRNA network was drawn with the help of Cytoscape 2.8.2. *P*<0.05 was used as the criterion for statistical significance.

### Statistical analysis

All statistical analysis in this study were performed by the Statistical Product and Service Solutions (SPSS) 16.0 software package (IBM, Chicago, IL) and GraphPad Prism 6.0 (GraphPad Software, La Jolla, CA). Paired *t* test, independent *t* test and one way analysis of variance (ANOVA) were used in this study correctly. A receiver operating characteristic (ROC) curve was established to value the diagnostic power. *P* value of 0.05 or less was considered statistically significant.

## Abbreviations

AFP: α-fetoprotein
AKP: alkaline phosphatase
ALT: alanine aminotransferase
ANRIL: antisense noncoding RNA in the INK4 locus
ANOVA: one way analysis of variance
AST: aspartate transaminase
BCLC: Barcelona clinic liver cancer staging system
Cdr1as: CDR1 antisense RNA
ceRNA: competing endogenous RNA
circRNAs: circular RNAs
Ct: threshold cycle
GGT: gamma glutamyl transferase
INK4/ARF: cyclin-dependent kinase inhibitor 2/ alternate open reading frame
miRNA: microRNA
MREs: miRNA response elements
ncRNA: non-coding RNA
qPCR: quantitative polymerase chain reaction
PCR: polymerase chain reaction
ROC: receiver operating characteristic
RT: reverse transcription
SMYD4: SET and MYND domain 4
SPSS: statistical program for social sciences
TP: total protein
TNM: tumor-node-metastasis
